# *Bacillus subtilis* 8–32 enhances tomato growth and reshapes rhizosphere microbial communities: insights into plant-microbe interactions

**DOI:** 10.3389/fpls.2026.1726342

**Published:** 2026-03-11

**Authors:** Bo Chen, Menghan Wang, Tongguo Gao, Hongquan Liu, Xiaoyu Wang, Yu Bai, Junpo Jiang, Baocheng Zhu, Dongdong Zhang

**Affiliations:** 1 Hebei Engineering Research Center for Resource Utilization of Agricultural Waste, College of Life Sciences, Hebei Agricultural University, Baoding, China; 2 State Key Laboratory of Crop Improvement and Regulation in North China, Hebei Agricultural University. North China Key Laboratory of Water Saving Agriculture, Ministry of Agriculture and Rural Affairs, Baoding, China

**Keywords:** *Bacillus subtilis*, colonization, growth promotion, microbial community, tomato

## Abstract

**Introduction:**

Plant growth-promoting rhizobacteria (PGPR) are crucial for sustainable agriculture, but their efficacy depends heavily on their colonization capacity within the rhizosphere and their interactions with native microbial communities. In previous studies, *Bacillus subtilis* strain 8-32 was screened for its potent antagonistic activity against pathogenic fungi and its high capacity for producing indole-3-acetic acid (IAA). This study aimed to investigate the colonization dynamics of strain 8-32 in tomato plants and evaluate its impact on the rhizosphere microbial community structure.

**Methods:**

Green fluorescent protein (GFP) labeling was used to track the colonization of *B. subtilis* 8-32 in tomato plants. Tomato seedlings were randomly divided into three treatment groups: the control group (CK), the seed-soaking group (T1), and the root-drenching group (T2). During the 21-day experimental period, plant growth parameters, root activity, and malondialdehyde (MDA) content were monitored. Changes in bacterial and fungal community structures were analyzed via high-throughput sequencing of the 16S rRNA and ITS regions.

**Results:**

The results revealed that *Bacillus subtilis* 8-32 successfully colonized both the tomato root system and the surrounding soil. On days 14 and 21, the colonization levels in the root system reached 7.1130±0.0413 (log_10_ CFU/g), while in the soil, they were 6.4664±0.03620 (log_10_ CFU/g) and 7.111±0.0461 (log_10_ CFU/g), respectively. T1 group and T2 group exhibited significant growth improvements compared to CK. Specifically, on day 14, the root length, root weight, stem length, and stem weight of T2 group increased by 19.96%, 381.81%, 39.97%, and 145.33%, respectively, compared to CK. Root vitality in the T2 group was 39.77%, 177.24%, and 171.16% higher on day 7, 14, and 21, respectively, while malondialdehyde content decreased by 24.60%, 34.18%, and 71.34%, over the period. Microbial diversity analysis revealed that *Bacillus subtilis* 8-32 did not significantly alter the community α-diversity (P>0.05), but selectively reshaped the community composition: it enriched beneficial bacterial taxa such as Proteobacteria, Bacteroidota, and Actinobacteriota, enhanced the functional diversity of Ascomycota, and concurrently reduced the abundance of pathogenic fungi within Basidiomycota.

**Discussion:**

These findings confirm that *Bacillus subtilis* 8–32 exerts growth-promoting effects on tomatoes through efficient colonization, regulation of rhizosphere microecological structure, and synergistic enhancement of plant stress resistance. Application of this strain by root drenching exhibits promising potential in tomato production, offering a novel approach to reduce reliance on chemical fertilizers and pesticides and facilitate the development of sustainable agriculture.

## Introduction

The rapid advancement of modern agricultural technology has spurred interest in microbial fertilizers, particularly Plant Growth-Promoting Rhizobacteria (PGPR), which have emerged as a focal point of recent research (Fasusi et al., 2021). The rhizosphere, the dynamic interface between plant roots and soil, serves as a critical hub for energy and material exchange, hosting a diverse array of microorganisms and invertebrates. Recognized as one of the most biologically active regions on Earth, the rhizosphere harbors microbial communities that play a pivotal role in influencing plant growth, nutrient acquisition, and overall health (Philippot et al., 2013; Mendes et al., 2013; Zhang et al., 2018). PGPR, a group of beneficial microorganisms commonly found in the rhizosphere, enhance crop growth through multiple mechanisms. These microorganisms degrade organic matter in the soil through their metabolic activities, thereby increasing nutrient availability for plants. Additionally, PGPR secrete phytohormones that stimulate crop growth and development, leading to improved yields and enhanced resistance to diseases and abiotic stress (Wang et al., 2024). The mechanisms by which PGPR promote plant growth can be broadly categorized into direct and indirect pathways. Direct promotion involves the synthesis of plant hormones, such as auxins, cytokinins, and gibberellins, which regulate key physiological processes, including seed germination, growth, and flowering (Chen et al., 2010). PGPR also facilitate the conversion of complex organic compounds into essential nutrients, such as nitrogen (N), iron (Fe), and phosphorus (P), which are readily assimilable by plants, thereby enhancing nutrient uptake and promoting growth (Zhang et al., 2021). Indirect promotion, on the other hand. involves the production of antagonistic substances, such as antibiotics and cell wall-degrading enzymes, which suppress plant pathogens (Danish et al., 2024). Furthermore, PGPR can degrade soil pollutants, improving soil physicochemical properties and modulate the production of Reactive Oxygen Species (ROS) scavenging enzymes in plants, thereby reducing oxidative stress, and enhancing stress tolerance (Ahmad, 2012).

The beneficial effects of PGPR are primarily mediated through their interactions with plants, making their ability to colonize soil, plant roots, or root surface tissues essential for their functionality (Lugtenberg and Dekkers, 2010). The colonization process involves two key steps: chemotactic movement toward the rhizosphere and the formation of biofilms (Sasse et al., 2018). Chemotaxis in response to root exudates represents a critical initial step in the recruitment and colonization of PGPR (Feng et al., 2021). Plants attract beneficial bacteria by releasing signaling molecules from their roots, while soil microorganisms in the rhizosphere can produce secretions that influence PGPR movement (Zhang et al., 2014). Upon exposure to these signals, PGPR aggregate around the root system, adhere to the surface, and secrete adhesion factors, such as polysaccharide matrices, fibrin, and lipoproteins, leading to the formation of extracellular polymeric substance, commonly referred to as a biofilms (Feng et al., 2018). The biofilms contribute to the decomposition of organic matter and the release of essential nutrients, including nitrogen, phosphorus, and potassium, which are vital for plant growth (Bais et al., 2006; Bamford et al., 2023).

Soil microorganisms play a critical role in regulating soil physicochemical properties and influencing plant productivity, serving as vital indicators of soil health. In agricultural ecosystems, diverse microbial communities—including bacteria, fungi, and nematodes—coexist within a multi-trophic microfood web, particularly at the root-soil interface, known as the rhizosphere microenvironment (Fan et al., 2020a). These microorganisms impact crop health and yield both directly and indirectly. However, managing and controlling soil microbial populations remains a significant challenge (Fan et al., 2020b). The composition of bacteria and fungi in the soil fluctuates with plant growth, and agricultural practices can substantially alter microbial diversity (Dove et al., 2021). Research indicates that the inoculation of exogenous PGPR can modify the structure of soil microbial communities as well as soil physicochemical properties (Li et al., 2022; Mawarda et al., 2020). For instance, Li et al. (2023) demonstrated that the addition of PGPR enhanced the diversity of bamboo soil community without disrupting their original richness. Similarly, Sun et al. (2025) employed co-inoculation of PGPR and *rhizobia* to mitigate the saline-alkaline characteristics of alfalfa rhizosphere soil, resulting in increased organic matter content, nutrient availability, and the relative abundance of beneficial microorganisms, such as *Eocercomonas* and *Colpoda*. These finding underscore the importance of understanding the interactions between PGPR and rhizosphere microbial community, as well as the mechanisms by which PGPR influence their structure and function.

Previous studies have identified *Bacillus subtilis* 8–32 as a soil-derived strain with remarkable plant growth-promoting and pathogen-antagonistic activities. This strain synthesizes indole-3-acetic acid (IAA) and siderophores, both of which are well-documented plant growth-promoting substances. In addition, it harbors antagonistic genes encoding fengycin, subtilin, and iturin, among which iturin is recognized as its primary antimicrobial component. The strain exerts broad-spectrum antagonistic activity against phytopathogens, including *Fusarium oxysporum*, *Fusarium solani*, and *Phytophthora cactorum*. Specifically, it disrupts fungal hyphae and inhibits spore germination (Wang et al., 2019). Furthermore, the antifungal metabolites secreted by this strain exhibit excellent stability, with high tolerance to temperature fluctuations, pH variations, and ultraviolet (UV) radiation, as well as resistance to protease degradation (Jia et al., 2019). These characteristics collectively render *Bacillus subtilis* 8–32 a promising candidate for microbial fertilizer development. This study investigates the effects of *Bacillus subtilis* 8–32 on tomato cultivation, focusing on its root colonization potential and its impact on the rhizosphere soil microbial community. High-throughput sequencing techniques were employed to analyze the influence of the 8–32 strain on microbial community structure in the tomato rhizosphere. The findings have potential implications for reducing reliance on chemical fertilizers and pesticides in tomato production and support the broader application of microbial fertilizers in sustainable agricultural practices.

## Materials and methods

### Strains and plasmids

The experimental strain *Bacillus subtilis* 8–32 was isolated from soil samples collected around Chaka Salt Lake in Qinghai Province, China. The strain exhibited an indole-3-acetic acid (IAA) yield of 45.29 mg/L when cultured in Luria-Bertani medium (tryptone 10 g/L, NaCl 10 g/L, yeast extract 5 g/L, pH 7.0), and demonstrated antagonistic activity against pathogens, including *Fusarium oxysporum* (Wang et al.,2019). The *Fusarium oxysporum* used in this study was provided by Professor Wang Guanghua from the Northeast Institute of Geography and Agricultural Ecology, Chinese Academy of Sciences. Additionally, the pHT315-GFPmut3a shuttle plasmid, containing ampicillin and erythromycin resistance genes, was kindly provided by Professor Liu Changhong from Nanjing University. This plasmid enables the cloning and expression of exogenous genes in both Gram-negative (*Escherichia coli*) and Gram-positive (*Bacillus subtilis*) bacteria and encodes the green fluorescent protein variant GFPmut3a.

### Construction of *Bacillus subtilis* 8-32-GFP tagged strain

The pHT315-GFPmut3a plasmid was extracted from *Escherichia coli* using a plasmid extraction kit (DP103-02, Tiangen Biochemical Technology, Beijing, China) and introduced into competent *Bacillus subtilis* cells via electroporation, following the protocol described by Yi and Kuipers (2017). The electroporated bacterial suspension was plated onto nutrient agar (NA, Beef extract 3 g/L, peptone 10 g/L, NaCl 5 g/L, agar 15 g/L, pH 7.2–7.4) plates supplemented with 5 mg/L erythromycin. Transformants were selected and confirmed for fluorescence using a fluorescence microscope.

### Characteristics of the tagged strain 8-32-GFP

Growth Curve Analysis: The growth differences between the tagged strain 8-32-GFP and the wild-type strain 8–32 were evaluated by measuring growth curves. Single colonies of both strains were inoculated into 100 mL of nutrient broth (NB, Beef extract 3 g/L, peptone 10 g/L, NaCl 5 g/L, pH 7.2–7.4) medium, with 5 μg/mL of erythromycin added for the tagged strain. After overnight incubation at 37 °C with shaking at 180 r/min, 2 mL aliquots were transferred to 100 mL of fresh NB medium. The optical density at 600 nm was measured at 0, 4, 8, 12, 16, 20, 24, 28, and 32 hours.

Genetic Stability Assessment: The genetic stability of the tagged strain 8-32-GFP was evaluated using a continuous passage method. The strain was subcultured on NA medium for 10 generations, and a single colony from the 11th generation was streaked onto NA plates containing erythromycin to confirm stability.

Functional Characterization: IAA production and antagonistic activity against pathogenic fungi were compared between the wild-type and tagged strains. IAA concentration was quantified using the Salkowski colorimetric method after 48 hours of shaking incubation in LB medium. The antagonistic activity of the labeled strain 8–32 was verified by the dual culture assay (Zhu et al., 2023). A 0.5 cm-diameter plug of activated *Fusarium oxysporum* was inoculated at the center of the PDA plate (Potato 200 g/L, glucose 20 g/L, agar 15 g/L, natural pH). Subsequently, a single colony of *Bacillus subtilis* 8–32 and three colonies of 8-32-GFP were inoculated equidistantly on the plate at a distance of 2.5 cm from the center of the fungal plug. The plates were incubated at 28°C for 7 days, and the antagonistic phenotypes were then recorded and analyzed. *Bacillus subtilis* 8–32 served as control, with three biological replicates performed for each treatment.

### Tomato planting experiment

*Solanum lycopersicum* L. ‘Ruili’ (GPD Tomato 620355) was purchased from the Baoding Seed Market. Healthy and plump tomato seeds were surface-disinfected and soaked in sterile water for 6 hours, followed by incubation at 25 °C in darkness for 48 h. The seeds were then transplanted into pre-treated nutrient soil germination trays. Three treatment groups were set up: Control group (CK): no microbial agent applied; Seed soaking group (T1): seeds soaked in *Bacillus subtilis* 8-32-GFP broth (3×10^8^ CFU/mL) for 20 minutes; Root drenching group (T2): 10 mL of 8-32-GFP broth applied after planting. Thirty plants were assigned to each treatment as biological replicates, and all groups were cultured in a greenhouse at 25 °C. Plant growth parameters were monitored at 7, 14, and 21 days after sowing.

Soil samples were collected from Baoding City, Hebei Province, China, at a depth of 10–20 cm. The tested soil was a type of cinnamon soil. The physicochemical properties of the amended soil were as follows: pH 6.7 ± 0.1 (soil-to-water ratio 1:2.5, potentiometric method); total nitrogen 3.9 ± 0.08 g/kg; total phosphorus 2.0 ± 0.083 g/kg; total potassium 36.6 ± 0.01 g/kg; ammonium nitrogen 32.7 ± 0.04 mg/kg; available phosphorus 20.3 ± 0.02 mg/kg; available potassium 150.0 ± 0.014 mg/kg.

### Tomato growth indicators

Plant height and weight were recorded at 7, 14, and 21 days. At 14 days, root parameters (length, volume, surface area, branching number, and tip number) were assessed using a root scanner (ES7000H, Shenzhen Xingye Equipment Co., Ltd.). Chlorophyll content was measured using a chlorophyll meter (SPAD-502D Plus, Konica Minolta, Japan). Malondialdehyde content was determined spectrophotometrically (Hagège et al., 1995), and root vitality was evaluated using the TTC reduction method.

### Colonization of strain 8-32-GFP

At 7, 14, and 21 days after tomato sowing, six plants were randomly selected from each treatment group. After shaking off loosely adhering soil from the roots, the soil tightly attached to the root surface was brushed off using a sterile brush and designated as rhizosphere soil. For the determination of bacterial colonization in soil: 1 g of rhizosphere soil sample was weighed and subjected to analysis via the dilution plating method. For the assessment of root colonization in roots tissues: the roots were rinsed with sterile water, blotted dry with absorbent paper, and 1 g of root tissue sample was weighed and ground into a homogenate, followed by analysis using the dilution plating method. Aliquots of the above-mentioned dilutions were spread onto NA plates supplemented with erythromycin (5 mg/L). After incubation at 37 °C for 48 h, the number of fluorescently labeled bacterial colonies on the plates was counted (Gao et al., 2016).

### DNA extraction, PCR amplification and sequencing library construction from soil samples

To minimize the impacts of soil physicochemical properties and environmental factors, the 7-day samples were selected for determination. At 7 days after tomato sowing (DAS), three plants were randomly selected from each treatment group, and rhizosphere soil samples were collected using the method described above. The collected samples were then pooled to form a single composite sample. Each treatment was performed with three biological replicates, corresponding to three composite samples per treatment. Total genomic DNA of the microbial community was extracted according to the instructions of the E.Z.N.A.^®^ Soil DNA Kit (Omega Bio-tek, Norcross, GA, USA). The quality of the extracted genomic DNA was detected by 1% agarose gel electrophoresis, and the DNA concentration and purity were determined using a NanoDrop 2000 (Thermo Scientific, USA). The V3-V4 variable regions of the bacterial 16S rRNA gene were amplified using the 338F/806R primer pair, while the first internal transcribed spacer (ITS1 region) of fungi rDNA was amplified with the ITS1F/ITS2 primer pair. PCR amplification was conducted in a 20 μL reaction mixture containing: 4 μL of 5×TransStart FastPfu Buffer, 2 μL of 2.5 mM dNTPs, 0.8 μL each of forward and reverse primers (5 μM), 0.4 μL of TransStart FastPfu DNA Polymerase, and 10 ng of template DNA (volume adjusted with sterile water). The amplification protocol was: initial denaturation at 95 °C for 3 min, followed by 27 cycles of denaturation (95 °C, 30 s), annealing (55 °C, 30 s) and extension (72 °C, 30 s), with a final extension at 72 °C for 10 min. Products were held at 4 °C post-amplification (ABI GeneAmp^®^ 9700 PCR System). PCR products were separated by 2% agarose gel electrophoresis, recovered and purified using a DNA Gel Extraction Kit (PCR Clean-Up Kit, YuHua, China), then quantified via a Qubit 4.0 Fluorometer (Thermo Fisher Scientific, USA).

Libraries were constructed from the purified PCR products using the NEXTFLEX Rapid DNA-Seq Kit, following the steps: (1) adapter ligation; (2) magnetic bead screening to remove adapter dimers; (3) PCR amplification for library template enrichment; (4) magnetic bead recovery of PCR products to obtain the final libraries. High-throughput sequencing was performed on the Illumina NextSeq 2000 platform (Shanghai Majorbio Bio-pharm Technology Co., Ltd.).

### Sequencing results analysis

Sequencing data were analyzed using the biological cloud platform of Shanghai Meiji Bio-Medical Technology Co., Ltd.,The raw sequencing data has been uploaded to the NCBI (National Center for Biotechnology Information) and assigned the accession number PRJNA1353837. Operational Taxonomic Units (OTUs) were clustered at a 97% similarity threshold using the UCLUST algorithm in QIIME. Taxonomic classification was performed using the Ribosomal Database Project (RDP) classifier. Alpha diversity and microbial community composition were assessed. Principal Component Analysis (PCA) and Principal Coordinates Analysis (PCoA) were used for dimensionality reduction on the original dataset to effectively illustrate the similarities and differences among samples. The Kruskal-Wallis rank-sum test and Linear discriminant analysis Effect Size (LEfSe) method were applied for species abundance analysis. Co-occurrence network analysis was conducted using Spearman correlation, focusing on the top 50 abundant species with an absolute correlation coefficient > 0.5 and P< 0.05.

### Data analysis

All data were analyzed using SPSS v26.0 software (IBM Corp., New York, USA). Data are presented as the mean ± standard deviation (SD). The Shapiro–Wilk test (p > 0.05) and Levene’s test (p > 0.05) were used to verify the distribution characteristics of the data in all groups, and the results confirmed that all experimental data met the prerequisites for one-way analysis of variance (ANOVA). Significant differences between groups were evaluated by one-way ANOVA followed by Duncans multiple range test (DMRT) *post-hoc* test. A p-value < 0.05 was considered statistically significant, and p < 0.01 was regarded as highly significant.

## Results

### Construction of *Bacillus subtilis* 8–32 tagged strain

**Figure 1:**
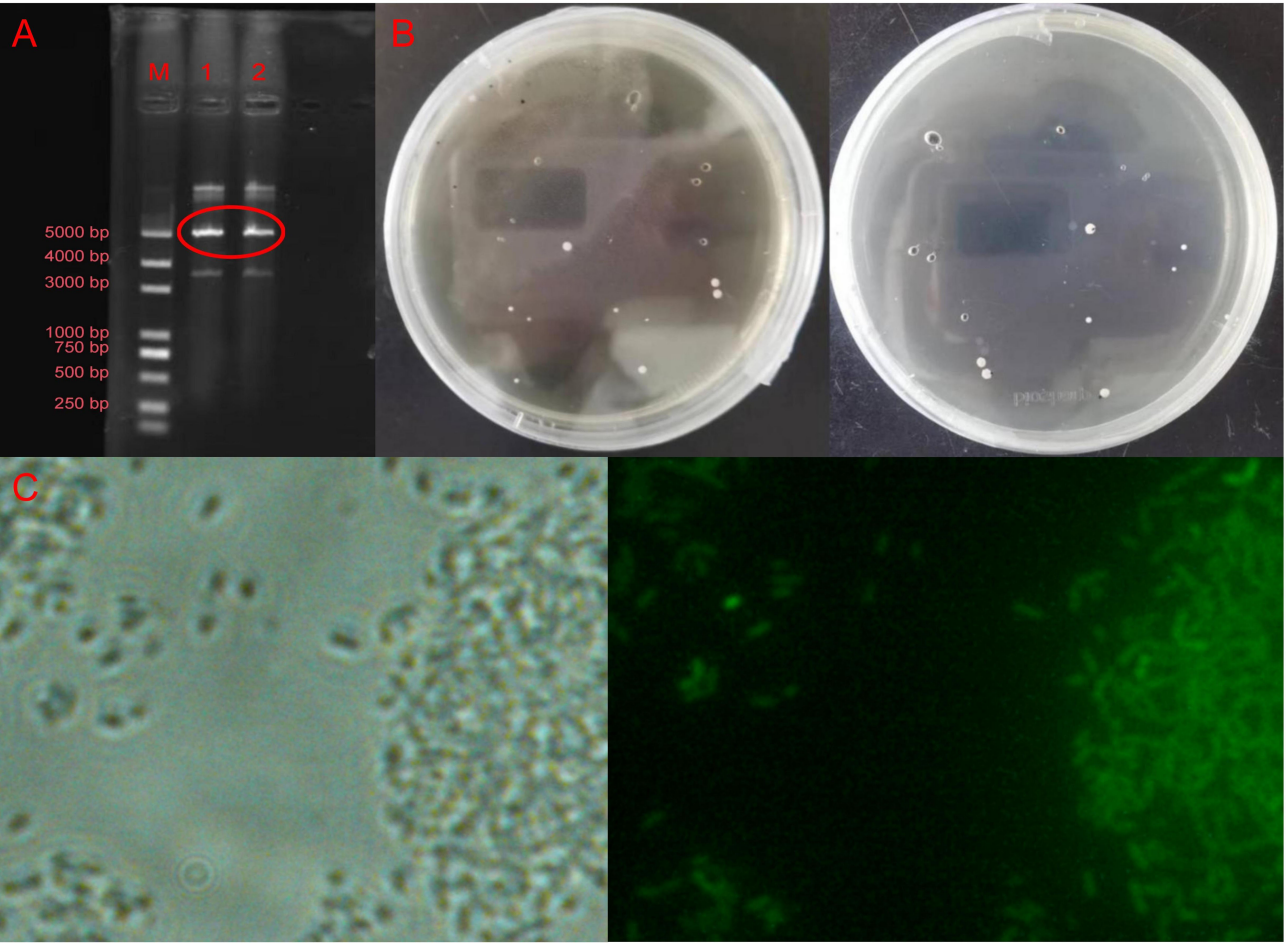
Construction of the labeled strain of *Bacillus subtilis* 8-32. **(A)** The pHT315-GFPmut3a extracted from *E.coli.* **(B)** Transformants in the NA resistance plate containing erythromycin. **(C)** Images of labeled cells under ordinary microscopy and fluorescence microscopy.

The pHT315-GFPmut3a plasmid (**Figure 1A**) was successfully transformed into *Bacillus subtilis* 8-32. Transformants were selected based on erythromycin resistance, as the wild-type strain was erythromycin-sensitive (**Figure 1B**). Furthermore, GFP expression in the transformed cells was confirmed by green fluorescence emission (**Figure 1C**).Utilize the M13 universal primer binding sites on the vector to amplify the inserted fragment via PCR and perform Sanger sequencing to verify the sequence accuracy.

### Characterization of *Bacillus subtilis* 8–32 tagged strains

**Figure 2:**
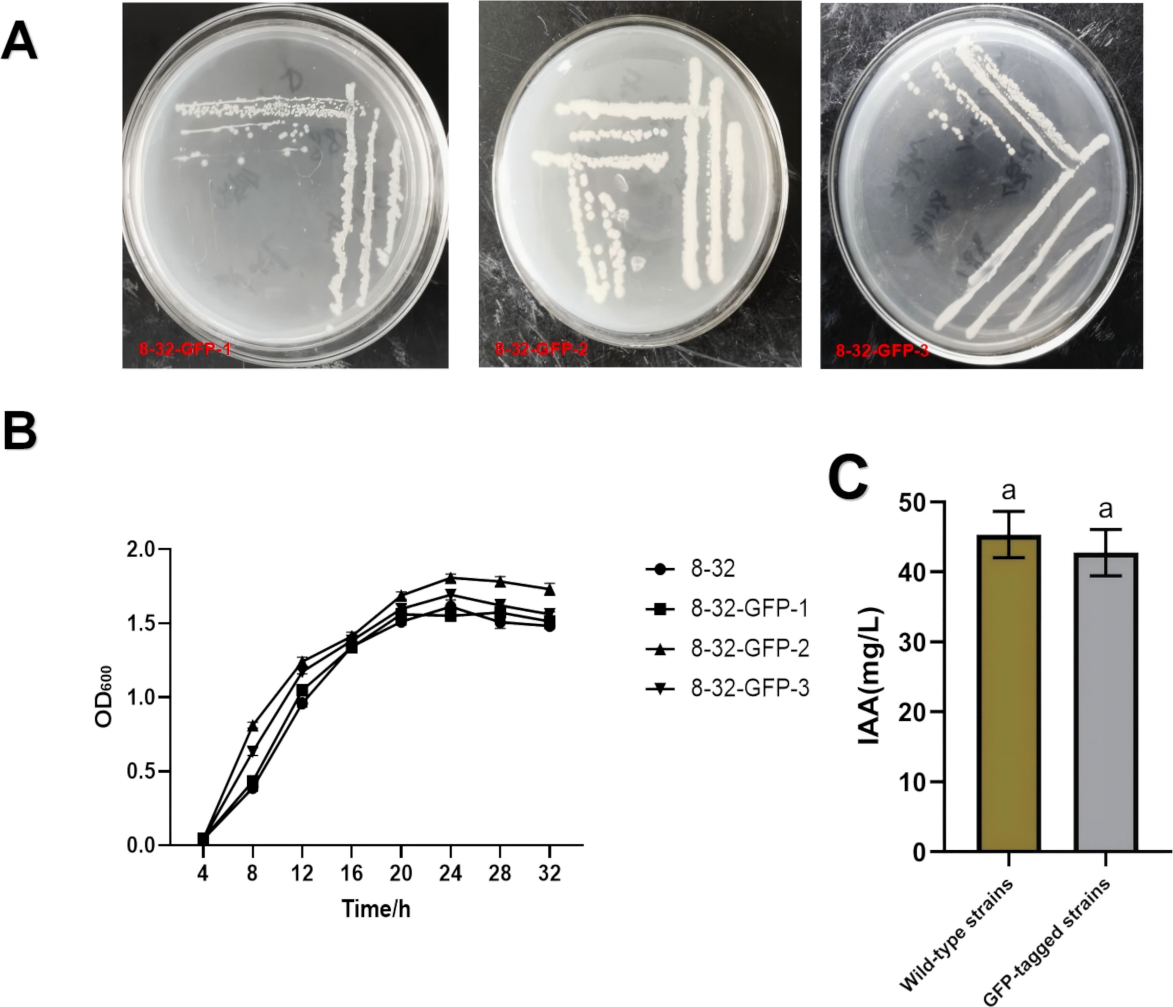
Analysis of the characteristics of the labeled strain of *Bacillus subtilis* 8-32. **(A)** Genetic stability of labled strains: a: First generation; b: Tenth generation; c: Eleventh generation. **(B)** Comparison of growth characteristics of labeled and wild-type strains. **(C)** Comparison of labled and wild-type strains in the production of indole-3-acetic acid (IAA).

Three transformants (8-32-GFP-1, 8-32-GFP-2, 8-32-GFP-3) were further analyzed. Growth curve analysis revealed no significant differences between the transformants and the wild-type strain, although 8-32-GFP-2 exhibited slightly superior growth performance (**Figure 2A**).

Genetic stability of the tagged strain 8-32-GFP was assessed through continuous passage in antibiotic-free medium. After ten generations, the strain maintained normal growth on erythromycin plates (**Figure 2B**), confirming stable plasmid replication. Comparative analysis of indole-3-acetic acid (IAA) production showed no significant difference between the wild-type (45.32 mg/L) and tagged strain (42.76 mg/L) (**Figure 2C**).

**Figure 3:**
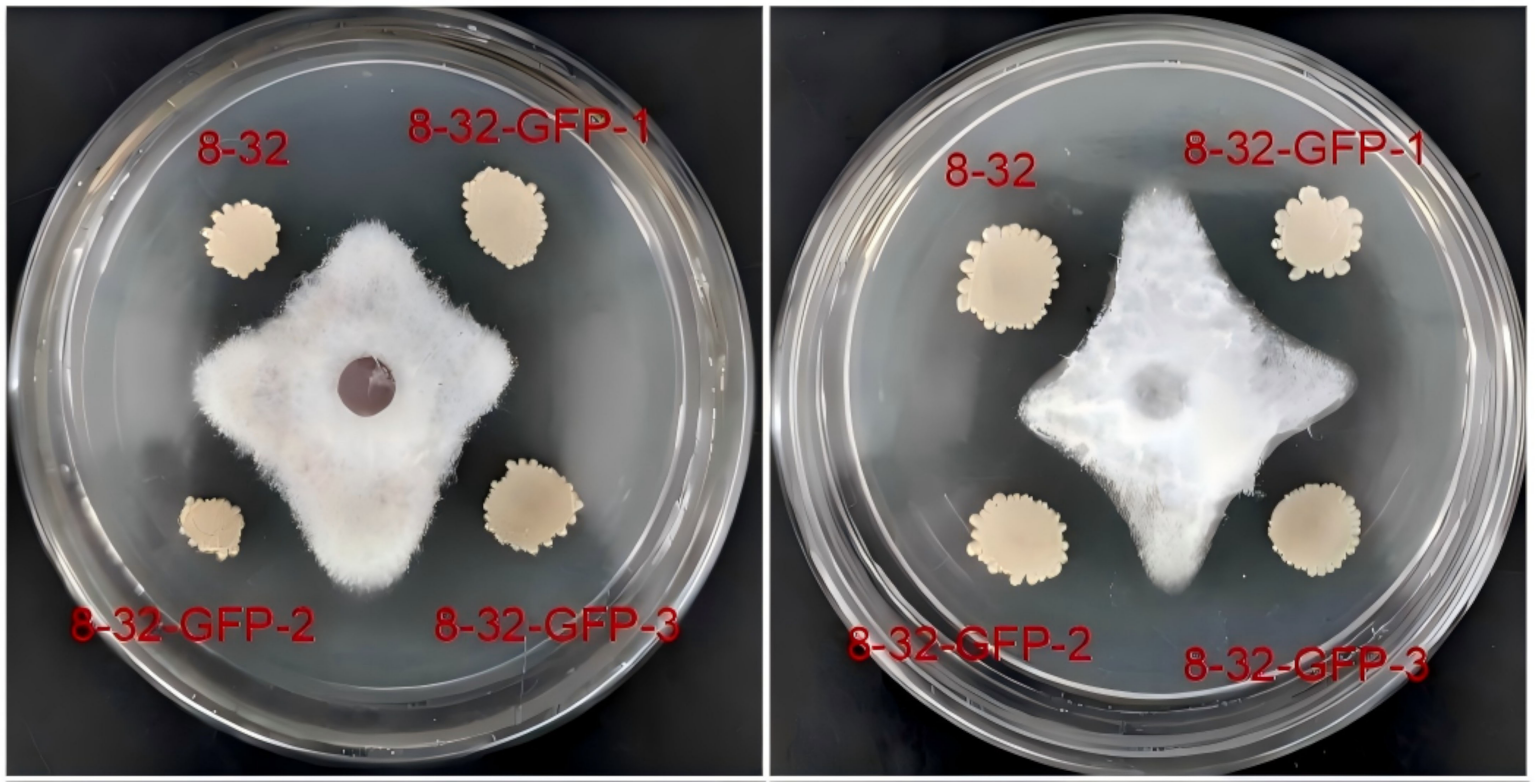
Comparison of antagonistic activities between labeled and wild-type strains against *fusarium oxysporum* by dual-culture plate method.

Furthermore, the antagonistic activities of the two strains against *Fusarium oxysporum* were confirmed to be similar through the dual culture assay (**Figure 3**). In the dual culture assay, the width of the inhibition zone of the wild-type strain 8–32 was 2.8 mm after 7 days of incubation, while that of the three labeled strains were 2.7 mm, 2.6 mm, and 2.8 mm, respectively, with no significant difference between the labeled strains and the wild-type strain.

### Colonization of *Bacillus subtilis* 8-32-GFP

**Figure 4:**
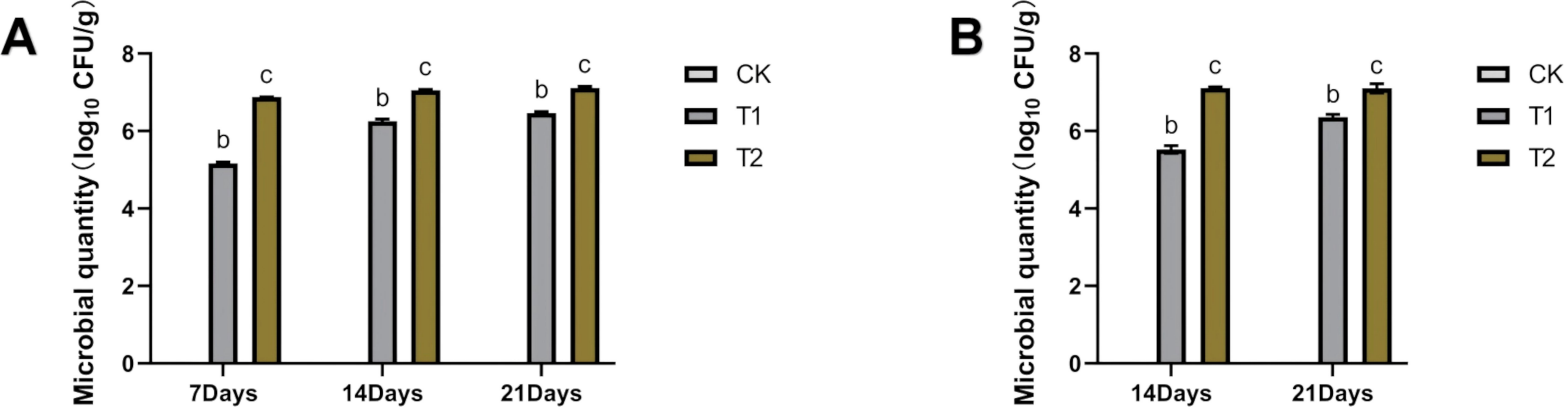
Colonization of the labeled strain in the roots of tomatoes and the rhizosphere soil. **(A)** Colonization in the rhizosphere soil of tomatoes. **(B)** Colonization in the roots of tomatoes. Footnote: No target viable bacteria were detected in the control group, and its log_10_ (CFU/g soil) was below 1.0 (limit of detection: 10 CFU/g soil).

Colonization of strain 8-32-GFP in tomato roots and rhizosphere soil was evaluated. Both T1 and T2 treatments enhanced colonization, with root colonization reaching 7.1130 ± 0.0413 (log_10_ CFU/g) and soil colonization reaching 6.4664 ± 0.03620 (log_10_ CFU/g) and 7.111 ± 0.0461 (log_10_ CFU/g)on days 14 and 21, respectively. By day 21, soil bacterial counts in T1 and T2 increased by 1895.91% and 71.63%, respectively, compared to day 7 (**Figure 4A**). Root colonization in T1 and T2 increased by 63.00% and 14.75%, respectively, from day 14 to day 21 (**Figure 4B**). Notably, roots colonization stabilized between days 14 and day 21.

### Tomato growth indicators

**Figure 5:**
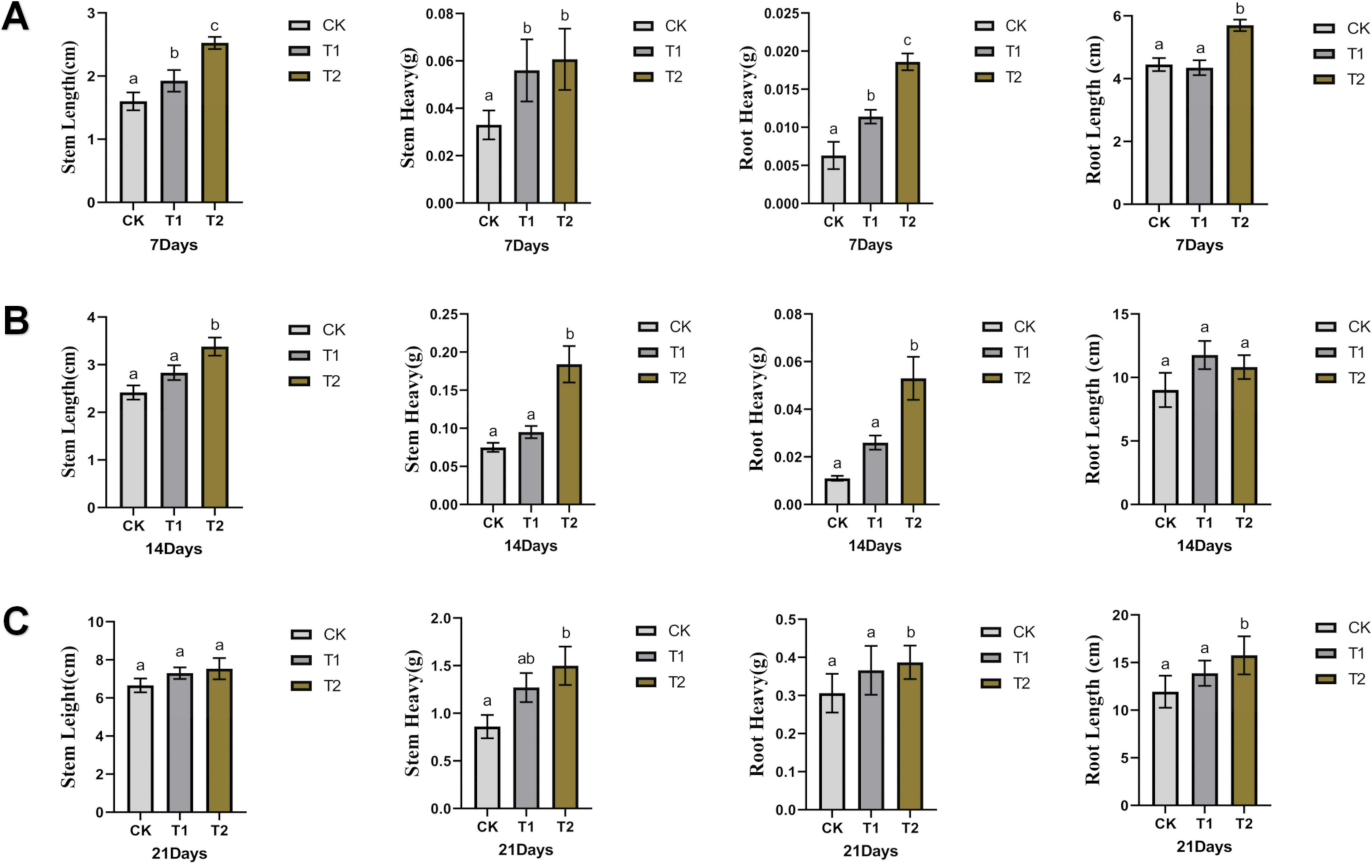
Influence of *Bacillus subtilis* 8-32 on Tomato Growth. **(A)** Stem leight, stem heavy, root heavy, and root lenght of tomato plants after 14 days of inoculation. **(B)** Stem leight, stem heavy, root heavy, and root lenght of tomato plants after 14 days of inoculation. **(C)** Stem leight, stem heavy, root heavy, and root lenght of tomato plants after 21 days of inoculation.

After 7 d of treatment with *Bacillus subtilis* 8-32 (**Figure 5A**), the growth parameters of tomato plants in all treatment groups were increased to varying degrees compared with the control group (CK). In the T1 group, underground fresh weight, stem length and aboveground fresh weight were significantly increased by 44.74%, 20.31% and 69.70%, respectively, with no significant difference in root length observed. The T2 group exhibited a more prominent growth-promoting effect, with root length, underground fresh weight, stem length and aboveground fresh weight increasing by 28.09%, 195.24%, 57.81% and 94.56%, respectively, relative to the CK group.

At 14 d post-treatment (**Figure 5B**), the growth advantages of the plants were further manifested. In the T1 group, root length, underground fresh weight, stem length and aboveground fresh weight were increased by 30.50%, 136.36%, 17.21% and 26.67% compared with the CK group, respectively. The T2 group showed a more remarkable increase in all parameters, reaching 19.96%, 381.81%, 39.97% and 145.33%, respectively, with the underground fresh weight presenting the most prominent increment.

**Figure 6:**
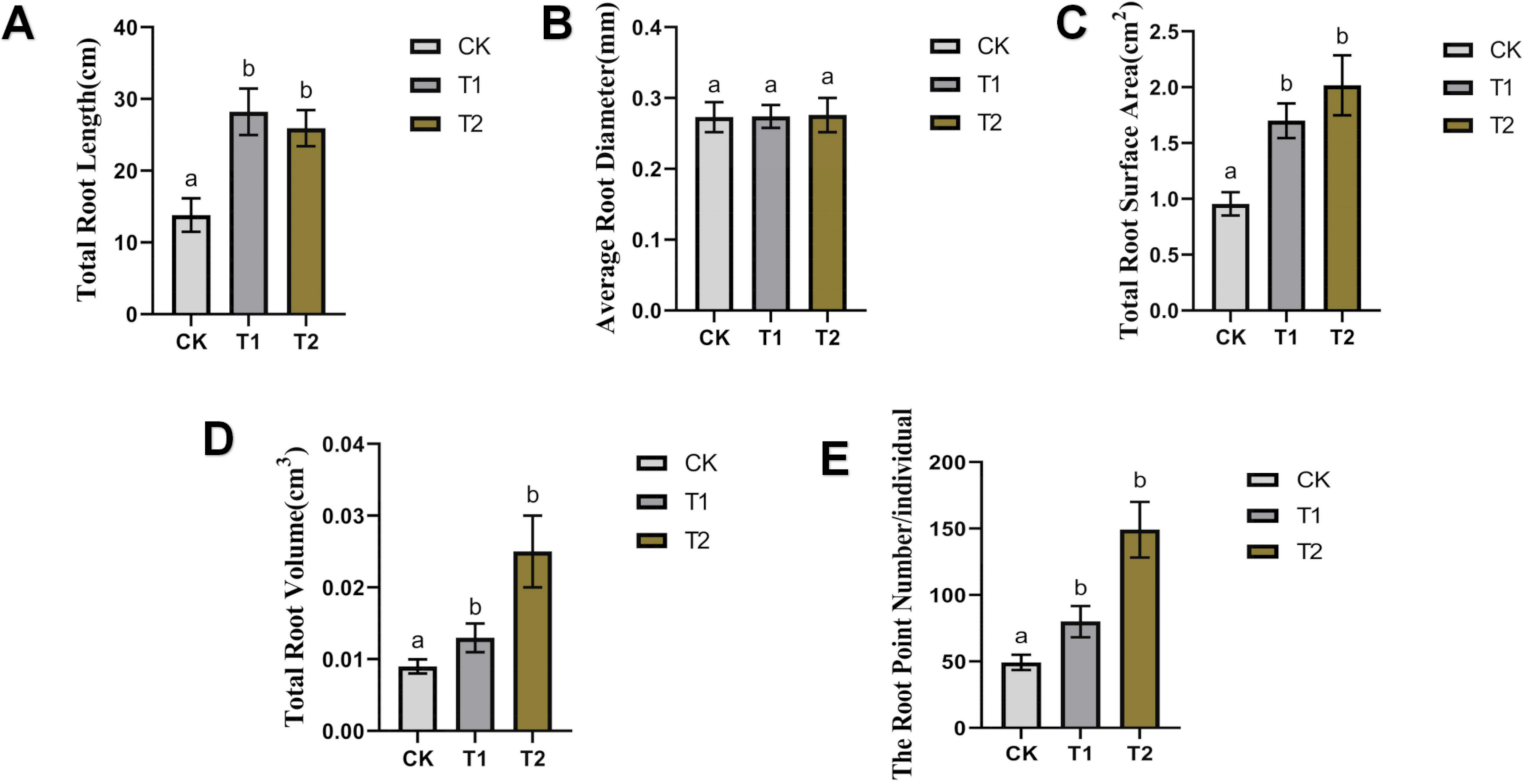
Scanning outcomes of tomato roots on day 14. **(A)** Total Root Length. **(B)** Average Root Diameter. **(C)** Total Root Surface. **(D)** Total Root Volume. **(E)** The Root Point Number.

By 21 d of treatment (**Figure 6C**), the growth parameters of both groups were continuously optimized. In the T1 group, the aforementioned parameters were further increased by 16.25%, 19.61%, 9.60% and 47.67% compared with those at 14 d. The T2 group maintained a superior growth trend, with the respective increments of these parameters reaching 31.99%, 26.47%, 13.21% and 74.30% relative to the 14 d level (**Figure 5C**).

### Root scanning results

Root scanning on day 14 revealed significant improvements in root architecture in both T1 and T2 treatments compared to CK (**Figure 6**). T1 increased total root length, surface area, volume, and root tips by 104.35%, 78.01%, 44.44%, and 62.16%, respectively, while T2 showed increases of 87.83%, 111.20%, 177.77%, and 202.36%, respectively. No significant differences in average root diameter were observed between treatments.

T1 increased total root length (**Figure 6A**), surface area (**Figure 6C**), volume (**Figure 6D**), and root tips (**Figure 6E**) by 104.35%, 78.01%, 44.44%, and 62.16%, respectively, while T2 showed increases of 87.83%, 111.20%, 177.77%, and 202.36%, respectively. No significant differences in average root diameter (**Figure 6B**) were observed between treatments.

### Chlorophyll content, malondialdehyde content, and root vitality

**Figure 7:**
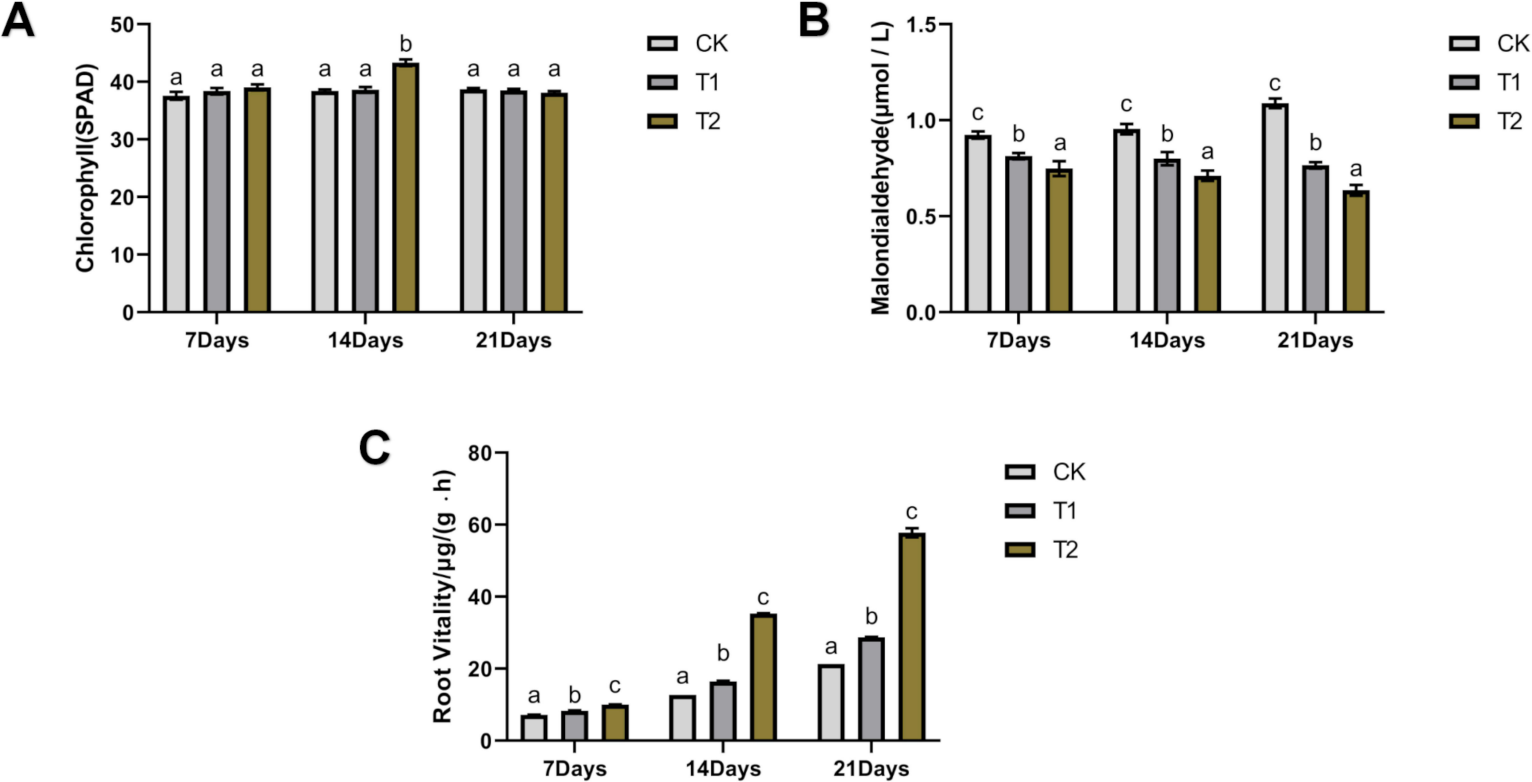
Effects of *Bacillus subtilis* 8–32 on the chlorophyll, malondialdehyde and root vitality. **(A)** Chlorophyll. **(B)** Malondialdehyde. **(C)** Root Vitality.

Chlorophyll content in tomato leaves was significantly higher in T2 compared to CK, with the increases of 3.89% and 12.76% at days 7 and 14, respectively (**Figure 7A**). T1 showed no significant difference from CK. Malondialdehyde (MDA) levels in leaf tissues were significantly reduced in both treatments, with T2 showing the most pronounced effect (p<0.05) (**Figure 7B**). MDA content in T1 decreased by 14.63%, 19.25%, and 42.22% at days 7, 14, and 21, respectively, while T2 showed reductions of 24.60%, 34.18%, and 71.34%, respectively. Root vitality was significantly enhanced in both treatments, with T2 exhibiting the highest levels (**Figure 7C**). T1 increased root vitality by 15.90%, 29.16%, and 34.86% at days 7, 14, and 21, respectively, while T2 showed increases of 39.77%, 177.24%, and 171.16%, respectively.

### Soil microbial alpha diversity analysis

The Shannon index and Simpson index were employed to evaluate the diversity of the microbial community. Higher values of these indices indicate greater microbial diversity. The variations in Alpha diversity indices of soil microorganisms across different treatments are shown in **Table 1**. The library coverage of samples from each treatment group exceeded 99%.

**Table 1:** Alpha diversity index.

Index	Ace	Chao	Coverage	Shannon	Simpson	Sobs
Bacteria						
CK	528.8 ± 11.24^a^	521 ± 19.06^a^	0.999 ± 0	3.81 ± 0.1455^a^	0.0579 ± 0.0127^a^	481 ± 2.646^a^
T1	514.4 ± 11.13^a^	501.5 ± 10.91^a^	0.999 ± 0	3.689 ± 0.1453^a^	0.0673 ± 0.0149^a^	473.7 ± 17.01^a^
T2	557.8 ± 49.16^a^	553.3 ± 43.36^a^	0.999 ± 0	3.929 ± 0.0142^a^	0.0496 ± 0.0015^a^	499 ± 46.87^a^
Fungi						
CK	65.38 ± 4.521^a^	63.56 ± 5.469^a^	0.999 ± 0	2.055 ± 0.0193^a^	0.2078 ± 0.0142^a^	61 ± 3.606^a^
T1	67.42 ± 8.621^a^	71.46 ± 13.02^a^	0.999 ± 0	2.0613 ± 0.1412^a^	0.2253 ± 0.0338^a^	61.33 ± 6.11^a^
T2	63.36 ± 6.54^a^	59.25 ± 6.222^a^	0.999 ± 0	1.849 ± 0.1351^a^	0.2654 ± 0.0457^a^	55 ± 2^a^

The diversity of a community can be evaluated using the Shannon index and the Simpson index. A lower value on the Shannon index, coupled with a higher value on the Simpson index, signifies reduced community diversity and an increased dominance of specific species. Additionally, the Chao1 index and the ACE index are employed to assess community richne.

number; means in a raw with different letters were significantly different (p < 0.05).

Analysis of microbial alpha diversity revealed no statistically significant differences in alpha diversity indices (e.g., Chao1, Shannon, and Simpson) among all treatment groups (P > 0.05). Specifically, the T2 group exhibited relatively higher bacterial richness and relatively lower fungal richness, this pattern merely reflected a distribution trend in community structure without reaching a statistically significant level.

### Bacterial community structure and composition

**Figure 8:**
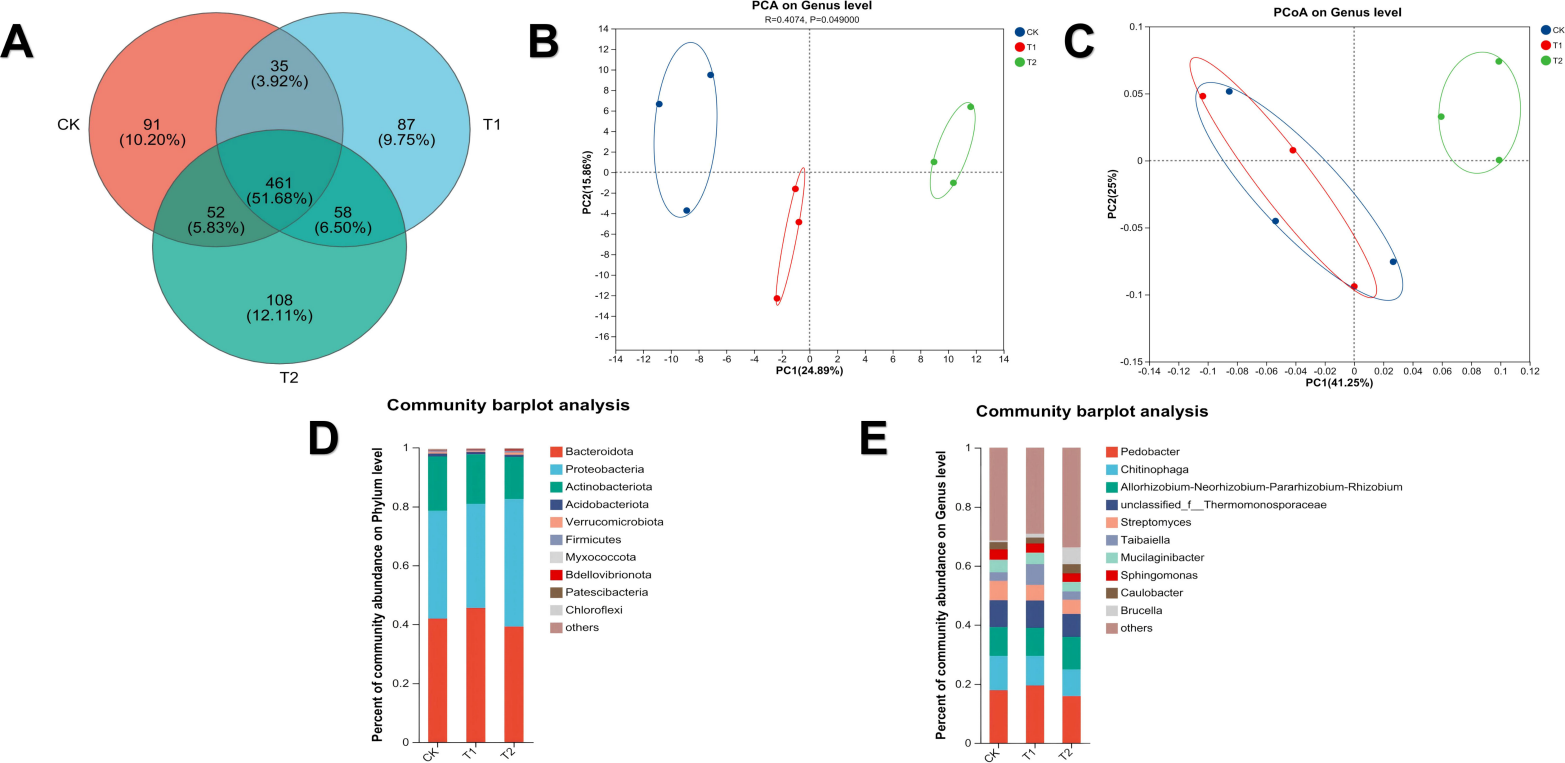
Bacterial community structure and composition in soil samples. **(A)** Species Venn diagram. **(B)** Soil PCA analysis. **(C)** Soil PCoA analysis. **(D)** Soil bacterial community composition at the phylum level. **(E)** Soil bacterial community composition at the genus level.

The species Venn diagram (**Figure 8A**) revealed distinct bacterial community structures among treatment groups, with 461 shared OTUs accounting for over 60% of the total. The T2 group exhibited the highest number of unique species compared to CK and T1 groups. Beta diversity analysis (**Figures 8B, C**) confirmed differences in bacterial community structure, particularly between the T2 group and the other groups.

At the phylum level of the bacterial community (**Figure 8D**), the top three dominant phyla were Bacteroidota, Proteobacteria, and Actinobacteriota, accounting for 97–98% of the total sequences, with no significant differences in dominant phyla among the treatment groups. At the genus level of bacterial community composition (**Figure 8E**), there were significant differences in relative abundance between the T1 and T2 groups. The top 10 dominant bacterial genera were *Pedobacter*, *Chitinophage*, *Allorhizobium-Neorhizobium-Pararhizobium-Rhizobium* complex, unclassified-f-Thermomonosporaceae, *Streptomyce*s, *Taibaiella*, *Mucilaginibacter*, *Sphingomonas*, *Caulobacter*, and *Brucella*, accounting for 66-71% of the total sequences. Compared with the T2 group and CK group, the relative abundance of *Taibaiella* in the T1 group was significantly increased (p < 0.05). The relative abundance of *Brucella* in the T2 group was significantly higher than that in the other two groups (p < 0.05).

### Fungal community structure and composition

**Figure 9:**
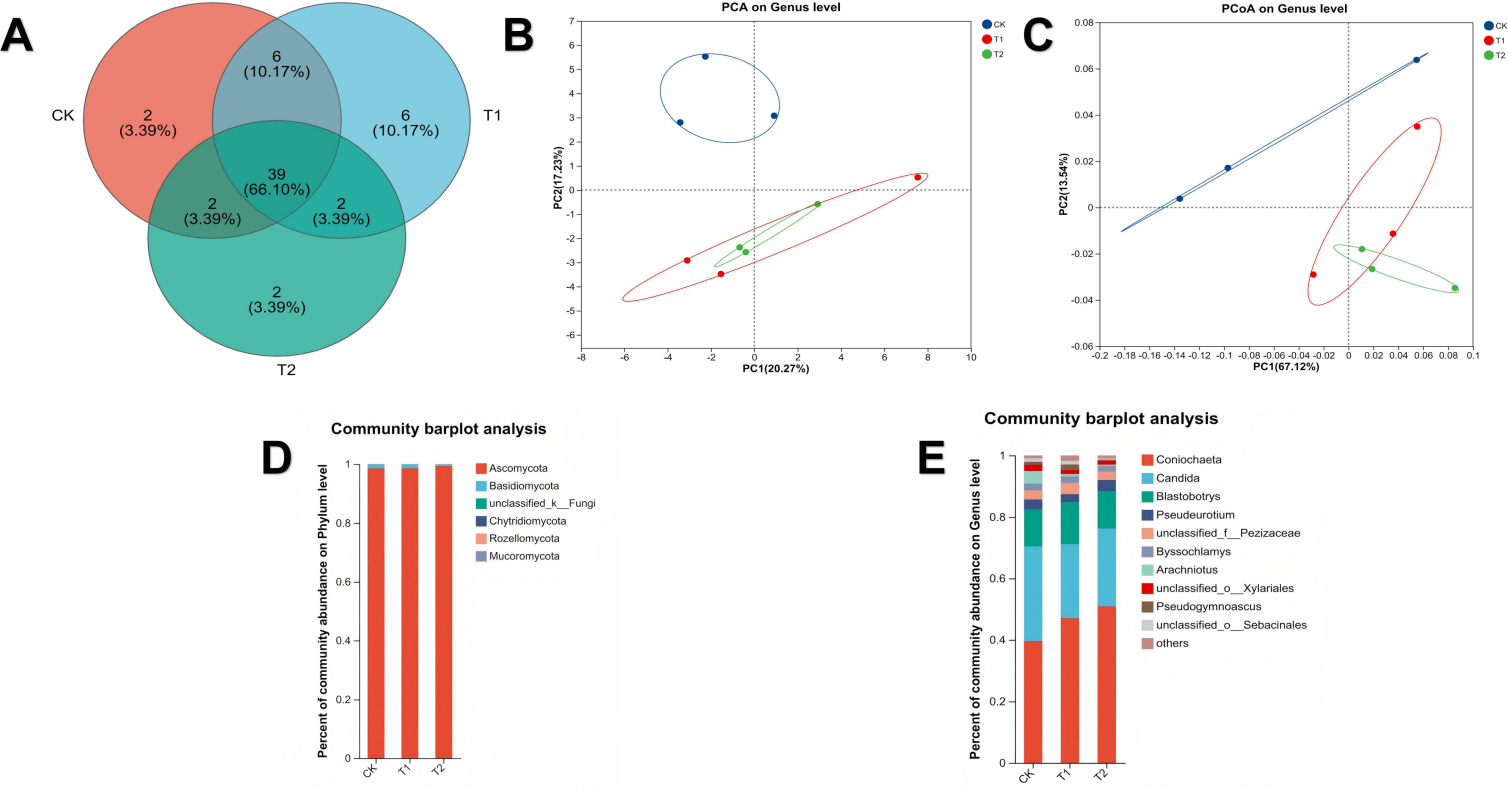
Fungal community structure and composition in soil samples. **(A)** Species Venn diagram. **(B)** Soil PCA analysis. **(C)** Soil PCoA analysis. **(D)** Soil fungal community composition at the phylum level. **(E)** Soil fungal community composition at the genus level.

The Venn diagram of fungal species (**Figure 9A**) showed a high degree of similarity among all groups, with 39 shared OTUs accounting for more than 70% of the total. Compared with the T2 group, both the CK group and T1 group exhibited higher species diversity. The CK group and T1 group had the highest similarity, and the T1 group harbored the most unique species. Beta diversity analysis (**Figures 9B, C**) revealed a significant clustering pattern between the T1 and T2 groups: along the principal component 1 (PC1) axis, samples from the CK group and T1 group were closely clustered near the 0 axis, while the T2 group showed significantly greater dispersion. The samples were relatively concentrated in the direction of PC1, whereas the separation between the T1 group and T2 group was extremely low in the direction of principal component 2 (PC2). Notably, the CK group was distinct from both the T1 and T2 groups along the PC2 axis. Principal Component Analysis (PCA) further confirmed that there were differences in the soil fungal community compositions among the three treatment groups, and their unique clustering patterns reflected the specific effects of different treatments on the microbial community structure.

At the phylum level of the fungal community (**Figures 9D**), Ascomycota was the core dominant phylum in all treatment groups, with a relative abundance accounting for 98%~99% of the total sequences, showing extremely high community dominance. The relative abundance of Basidiomycota in the T2 group was significantly lower than that in the CK group and T1 group (P<0.05), presenting obvious intergroup differences. Differential analysis at the fungal genus level showed (**Figure 9E**) that the relative abundances of two genera, *Coniochaeta* and *Pseudeurotium*, in the T2 group were significantly higher than those in the CK group and T1 group, respectively. In contrast, *Pseudogymnoascus* and *Arachniotus* were enriched in the T1 group and CK group, respectively, with significantly higher relative abundances (P<0.05). Additionally, the relative abundances of *Pseudogymnoascus* and unclassified-o-Sebacinales in the T2 group were significantly lower than those in the other two groups (P<0.05).

### Species difference analysis

The LDA discriminant plot displays the significantly differential species with an LDA score greater than the preset threshold, i.e., the statistically distinct biomarkers(p<0.05). Herein, the preset threshold is 2.0 (refer to the x-axis; only species with an absolute LDA value exceeding 2 are shown in the plot). The colors of the bars represent their respective groups, and the bar lengths correspond to the LDA scores, which indicate the magnitude of the impact of the significantly differential species among different groups.

**Figure 10:**
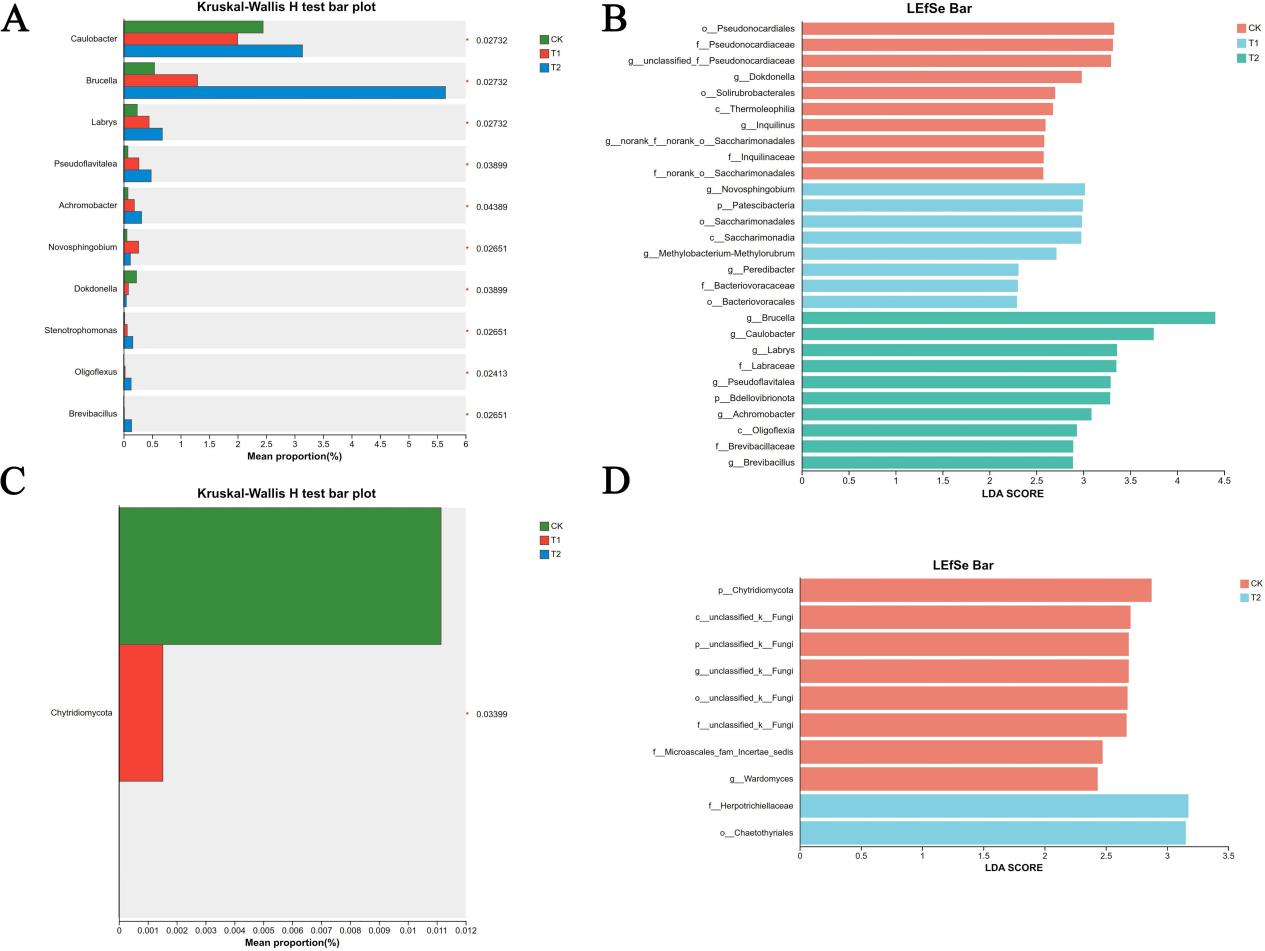
Comparison of species differences in different treatment soil samples. **(A)** Significance Test of Differences between Soil Bacterial Groups. **(B)** LDA Discriminant Results between Soil Bacterial Groups. **(C)** Significance Test of Differences between Soil Fungal Groups. **(D)** LDA Discriminant Results between Soil Fungal Groups.

Significant differences in bacterial genus abundances were observed (**Figure 10A**), with *Caulobacter*, *Brucella*, *Labrys*, and *Pseudoflavitalea* showing higher abundances in T2 group compared to the CK and T1 groups. Conversely, the abundances of *Novosphingobium* and *Dokdonella* were markedly lower in the T2 group than in both the CK and T1 groups. The LEfSe multi-level species difference analysis for tomato soil bacteria correspond to the LDA discrimination results shown in **Figure 10B**. The analysis revealed distinct variations in the relative abundances of bacterial taxa across the treatment groups. Notably, the T2 group eahibited the highest overall bacterial abundance, with *Brucella* and *Caulobacter* emerging as the dominant genera, while the CK had higher abundances of *Pseudomonas*, *Neosphingolipids*, and *Dokdonella* compared to both the T1 and T2 groups.

**Figure 10C** clearly showed significant variations in the fungal abundance across the three groups, with the CK group exhibiting the highest abundance at 0.113%, followed by the T1 group at 0.0441%, and the T2 group with the lowest fungal abundance at 0.01522%. The LEfSe multi-level species difference analysis for soil fungi corresponds with the LDA discrimination results presented in **Figure 10D**. The analysis revealed that the CK group was characterized by eight distinct fungal taxa that significantly contributed to inter-group differences, including Chytridiomycota. In contrast, the T2 group uniquely harbored Herpotrichiellaceae and Chaetothyriales, both belonging to the Ascomycota phylum, which were absent in the other groups.

### Co-occurrence network analysis

**Figure 11:**
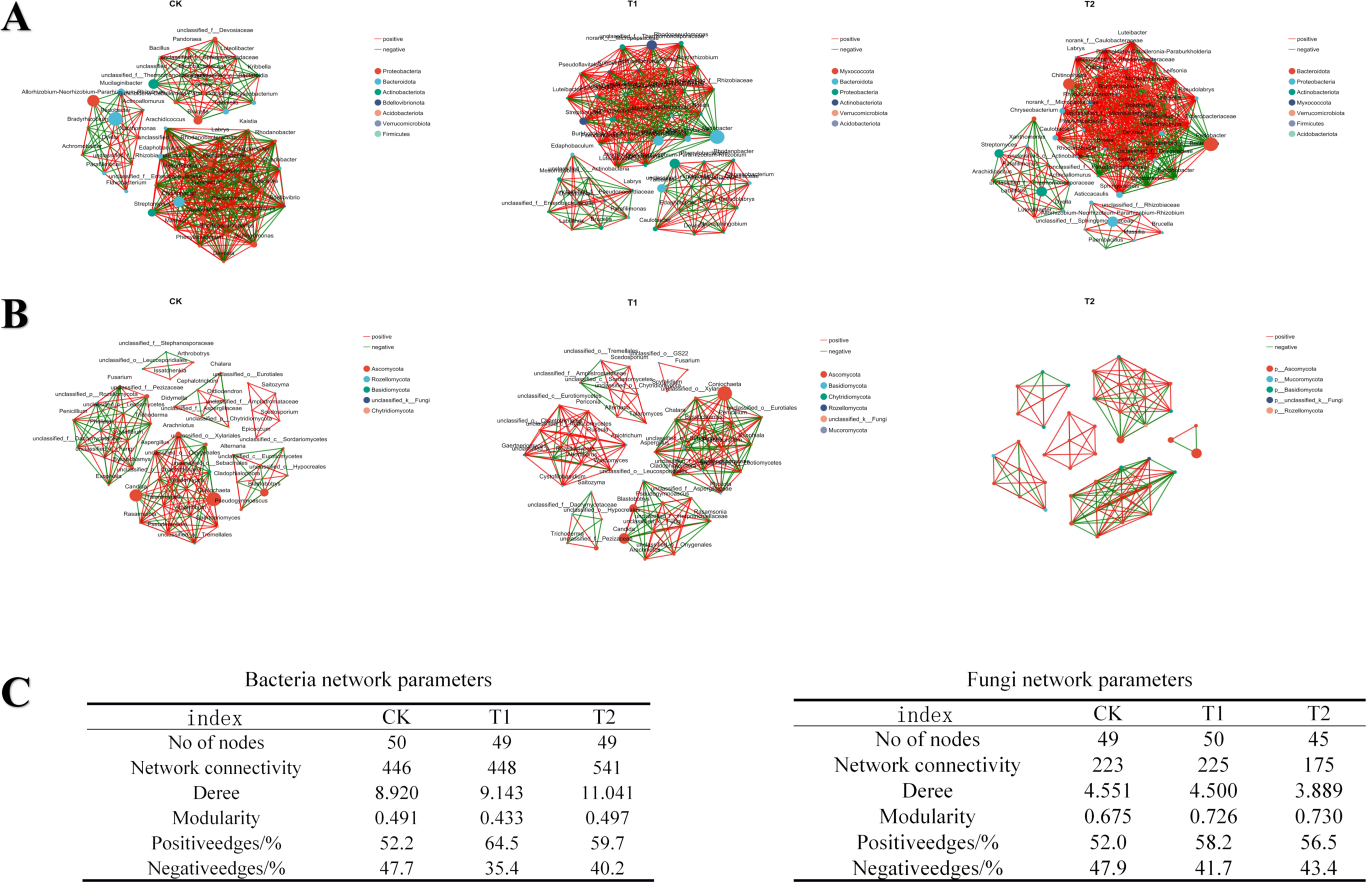
Co-occurrence networks among different treatment groups in tomato soil. **(A)** Bacterial Community Network. **(B)** Fungal Community Network. **(C)** Co-occurrence Network Parameters.Tables.

A co-occurrence network was constructed for the top 50 genera based on their abundance in tomato soil, using a defined threshold (correlation coefficient > 0.5, P < 0.05), as shown in **Figure 11**. The size of the nodes was proportional to the relative abundance of OTUs. In the bacterial correlation network diagram (**Figure 11A**) Proteobacteria was the most abundant bacterial phylum in the control group, while in the T1 and T2 groups, Bacteroidota and Proteobacteria exhibited higher relative abundances. The application of 8–32 strain significantly increased the abundance of both Bacteroidota and Proteobacteria within the network.

The fungal correlation network, illustrated in **Figure 11B**, demonstrated that Ascomycota displayed the highest species richness across all three treatment groups. This phylum encompasses several important fungal taxa involved in organic matter decomposition and nutrient cycling in soil ecosystems. Furthermore, the relative abundance of Mucoromycota was significantly higher in the T2 group compared to the CK and T1 groups.

Network complexity was evaluated based on the number of nodes, edges, and degrees within the co-occurrence network (**Figure 11C**). Compared to the CK group, the experimental groups (T1 and T2) exhibited an increase in the number of edges and degrees in the bacterial network, with the most pronounced changes observed in the T2 group. However, the number of nodes in the bacterial network remained relatively unchange. Conversely, the fungal network displayed a decline in the number of nodes, edges, and degrees. The application of *Bacillus subtilis* 8–32 resulted in increased complexity of the bacterial network while reducing the complexity of the fungal network. Additionally, the proportion of positive correlations between nodes in both bacterial and fungal co-occurrence networks increased. The modularity index, which reflects the extent of node clustering within the network (values > 0.4 indicate a modular structure), suggests that the network can perform specific ecological functions through distinct modules.

## Discussion

### Colonization mechanism of *Bacillus subtilis* 8–32 and its interaction with plants

Efficient colonization of plant growth-promoting rhizobacteria (PGPR) in the rhizosphere is a prerequisite for exerting their plant growth-promoting and biocontrol functions, and colonization efficiency depends on bacterial chemotaxis, biofilm formation, and signal crosstalk with plant roots (Lugtenberg and Dekkers, 2010). The GFP labeling method has emerged as a widely adopted tool in modern detection techniques due to its unique advantages. GFP can bind to various protein termini without altering the structural integrity or functionality of the original protein. Additionally, it exhibits excellent stability, enables straightforward detection without the need for complex sample processing, and allows for dynamic real-time tracking of live cells (Sheen et al., 2010). For instance, Cao et al. (2011) used the shuttle vector pHARII to label *Bacillus subtilis* SQR9, successfully monitoring its colonization and persistence in the cucumber rhizosphere, as well as on the surface of the primary root and at the junctions of lateral roots. Wang et al. (2020) employed GFP to label *Bacillus cereus* YL6 to investigate the physiological regulatory mechanisms of plant phosphate-solubilizing bacteria. Their findings confirmed that the GFP marker did not affect the physiological and biochemical characteristics of strain YL6, enabling the successful observation of its dynamic colonization process in Chinese cabbage. This study verified by GFP labeling that *Bacillus subtilis* 8–32 stably colonized tomato roots and rhizosphere soil Relevant studies have shown that *Bacillus* species can secrete adhesion factors (polysaccharide matrix, lipoproteins) to form biofilms, thereby enhancing their binding capacity on plant root surfaces; Meanwhile, they utilize organic acids and amino acids in root exudates to maintain population sizes, and further establish a strain-root-soil microecological interface (Chen et al., 2012; Pomerleau et al., 2024). Notably, root drenching exhibited better colonization performance than seed soaking, owing to the direct colonization pathway: root drenching allows direct root contact and avoids soil-induced activity inhibition during seed germination, while seed soaking relies on strain migration to rhizosphere and suffers from soil particle adsorption and indigenous microbial competition (Feng et al., 2021). Subsequent experiments demonstrated that the tagged strains maintained genetic stability and biological functions after subculture, indicating that the introduction of the pHT315-GFPmut3a plasmid did not affect their core metabolism (Dong et al., 2022; Kang et al., 2018), thus ensuring long-term rhizosphere colonization which ensures long-term rhizosphere colonization (Wang et al., 2020). Although no significant differences were observed in growth kinetics and colonization efficiency between the GFP-tagged strains and the wild-type strains, the heterologous expression of fluorescent proteins may impose a metabolic burden on host cells by competing for biosynthetic resources (Matsuyama et al., 2024). While multiple studies have demonstrated that the expression of GFP at an appropriate level exerts a negligible effect on the reproduction and metabolism of microorganisms (Binet et al., 2018; Scott et al., 2000), the possibility of subtle alterations in the bacterial metabolome cannot be completely ruled out. Therefore, the uncharacterized metabolic shifts potentially induced by GFP marker gene expression remain a limitation of this study. Future studies are recommended to employ metabolomic approaches for the comprehensive evaluation of marker gene-induced effects on the metabolite profiles of *Bacillus subtilis* 8-32.

These findings highlight the effectiveness of GFP labeling for tracking PGPR colonization, and underscore the potential of *Bacillus subtilis* 8–32 as a potent PGPR strain for enhancing plant-microbe interactions.

### Growth-promoting mechanism of *Bacillus subtilis* 8-32: growth regulation

Indole-3-acetic acid (IAA), a key regulator of plant growth and development, is synthesized by rhizobacteria and endophytic bacteria as one of their primary mechanisms for promoting plant growth (Duca et al., 2014). Safiyh et al. (2009) demonstrated that inoculation with IAA-producing strains significantly enhanced poplar growth, increasing biomass by 60% compared to the control group. *Bacillus subtilis* 8-32, a well-characterized plant growth-promoting bacterium, is notable for its high IAA production capacity. However, the growth-promoting effect of *Bacillus subtilis* 8–32 on tomato cannot be attributed to a single mechanism. First, the strain’s high indole-3-acetic acid (IAA) production capacity is the core driver of growth promotion: as a key plant hormone, IAA regulates cell division and elongation in the root apical meristem to increase root length, root surface area and lateral root number (Duca et al., 2014). In this study, the root fresh weight and root tip number of the T2 group were increased by 381.81% and 202.36%, respectively, which represents a direct manifestation of root morphogenesis regulated by IAA. Second, the strain enhanced plant stress tolerance by reducing malondialdehyde (MDA) content in tomato leaves (with a maximum reduction of 71.34%) and increasing root activity (with a maximum increase of 177.24%). As a key metabolite, MDA serves as an indicator of oxidative damage and membrane lipid peroxidation, and its level is affected by environmental stress, oxidative damage and metabolic disorders (Qin et al., 2016). For instance, Campos et al. (2003) reported that cold stress significantly increased MDA levels in plant cells, leading to the aggregation of biological macromolecules and membrane damage, thereby disrupting biochemical reactions. Similarly, Sapre et al. (2018) demonstrated that inoculation with the salt-tolerant plant growth-promoting strain IG3 under 100 mmol/L NaCl stress significantly decreased antioxidant enzyme activity and MDA content compared with the non-inoculated control group. The reduction in MDA content indicates alleviated membrane lipid peroxidation, while the increase in root activity enhances the absorption efficiency of water and nutrients. Collectively, these two effects mitigate the damage of abiotic stress to plants, a mechanism closely associated with the ability of *Bacillus subtilis* to activate the plant antioxidant enzyme system and scavenge reactive oxygen species (ROS) (Bi et al., 2024; Qin et al., 2016). Notably, the growth-promoting effect of the T2 group was significantly superior to that of the T1 group. Beyond the difference in colonization abundance, this may be due to the root drenching treatment enabling the strain to act directly on the root absorption zone and initiate hormone synthesis and nutrient activation pathways more rapidly. In addition, studies have shown that PGPR application can significantly increase chlorophyll content in plants (Yan et al., 2024). In this study, a significant increase in chlorophyll content was observed in tomato plants treated with *Bacillus subtilis* 8-32.

### Regulatory mechanism of the rhizosphere microbial community: selective enrichment and functional optimization

Rhizosphere soil microorganisms are among the most dynamic components in soil ecosystems, playing a pivotal role in maintaining soil health and promoting crop growth. These microorganisms are integral to various biochemical processes, including organic matter decomposition, humus formation, nutrient cycling, and ecosystem stabilization under environmental fluctuations (Prashar et al., 2014). The structure of soil microbial communities varies significantly across different ecosystems and microhabitats, with bacteria and fungi generally dominating both biomass and diversity, often exceeding other microbial groups by several orders of magnitude. Therefore, understanding the shifts in microbial diversity and community composition during tomato growth, particularly in response to *Bacillus subtilis* 8-32, is essential for elucidating its growth-promoting mechanisms.

This study found that *Bacillus subtilis* 8–32 did not significantly alter microbial α-diversity (P>0.05), indicating that the exogenously applied *Bacillus subtilis* 8–32 had no remarkable impact on the overall diversity of the soil microbial community, and the species richness and evenness of the soil microecosystem remained relatively stable. This characteristic demonstrated that during colonization and functional exertion in soil, strain 8–32 does not exert its effects by changing the overall structure of the microbial community, but rather tends to regulate the relative abundance of specific beneficial or harmful genera through interspecific competition and interaction, thereby reshaping the community composition structure. This is consistent with the results of significant differences in the abundance of specific genera among the treatment groups in this study.

In the bacterial community, this strain enriched beneficial taxa including Proteobacteria, Bacteroidota and Actinobacteriota, among which the abundances of *Brucella* (with nitrogen fixation function), *Caulobacte*r (growth-promoting metabolite secretion) and *Thermomonosporaceae* (antimicrobial substance production) were significantly increased (p<0.05). Proteobacteria comprises multiple classes such as α-,β-, andγ-Proteobacteria. Among them, *Brucella* spp. and the *Allorhizobium*-*Neorhizobium*-*Pararhizobium*-*Rhizobium* complex in α-Proteobacteria possess efficient nitrogen fixation capacity, and can convert atmospheric nitrogen into ammonia nitrogen via nitrogenase encoded by the *nifH* gene (Zhang et al., 2024). In addition, multiple taxa within Proteobacteria can secrete quorum sensing signal molecules (e.g., N-acyl homoserine lactones, AHLs), which form signal crosstalk with the AI-2 signal of *Bacillus subtilis* to regulate the group behavior of rhizosphere microorganisms, enhance biofilm formation efficiency and antimicrobial substance secretion (Bamford et al., 2023). This is also a key driving factor for the increased complexity of the bacterial network in the T2 group.

The enrichment of Bacteroidota primarily relies on its strong organic matter degradation capacity and environmental adaptability. Strains within this phylum generally contain abundant carbohydrate-active enzyme (CAZymes) gene clusters, which can efficiently degrade macromolecular organic substances such as cellulose, hemicellulose and pectin in soil into small-molecule sugars and organic acids that are easily absorbable and utilizable by plants and microorganisms (Xiao et al., 2024). In this study, dominant genera of Bacteroidota including *Pedobacter* and *Chitinophaga* showed high abundances, and the chitinase secreted by these genera can assist in degrading the cell walls of pathogenic fungi, forming a synergistic antibacterial effect with lipopeptide substances produced by *Bacillus subtilis* (Ongena & Jacques, 2008). Furthermore, Bacteroidota strains can enhance soil particle aggregation by secreting extracellular polysaccharides, improve soil aeration and water retention capacity, and create a suitable microenvironment for the colonization of *Bacillus subtilis* 8–32 and the growth of tomato roots, reflecting a dual regulatory mechanism of “functional complementarity-environmental optimization”.

The increased abundance of Actinobacteriota is crucial for strengthening rhizosphere biocontrol potential. Taxa such as *Thermomonosporaceae* and *Streptomyces* in this phylum can produce a variety of antimicrobial secondary metabolites including actinomycin and polymyxin, which exert broad-spectrum inhibitory effects on pathogenic fungi (e.g.,Basidiomycota) (Zhao et al., 2022). Compared with *Bacillus subtilis* 8-32, Actinobacteriota strains produce antimicrobial substances with longer persistence and a broader spectrum of action, and the two form a biocontrol synergistic network characterized by “short-term rapid antibacterial activity + long-term continuous prevention and control”. In addition, Actinobacteriota can chelate soil iron ions by secreting siderophores (e.g., bacillibactin), restricting nutrient acquisition by pathogenic microorganisms while simultaneously providing iron nutrition for plants to further enhance the growth-promoting effect (Zhang et al., 2024). In this study, the abundance of Actinobacteriota in the T2 group was significantly increased, which was causally associated with the decreased abundance of Basidiomycota pathogenic fungi, reflecting the core role of this phylum in the construction of the rhizosphere microecological barrier.

The synergistic effect of these three dominant phyla jointly promoted the functional optimization of the rhizosphere microecosystem. Additionally, the abundances of potential harmful taxa such as *Novosphingobium* and *Dokdonella* were reduced, which may be attributed to *Bacillus subtilis* 8–32 and the three dominant phyla jointly occupying the rhizosphere nutrient niche, or inhibiting their growth and reproduction by synergistically secreting antimicrobial substances (Fan et al., 2020b).

Colonization of *Bacillus subtilis* in crop roots altered the structure and diversity of the fungal community in rhizosphere soil, with an overall decreasing trend in fungal abundance. In the fungal community, the strain significantly reduced the abundance of Basidiomycota (P<0.05), which encompasses various plant pathogenic fungi such as *Peronosclerospora maydis* (corn downy mildew) and rust fungi (Brefort et al., 2009; Singh et al., 2011). The reduction in its abundance directly lowered the risk of soil-borne diseases. Meanwhile, Chytridiales and Arthrodermatales of Ascomycota were uniquely detected in the T2 group; these fungi possess efficient organic matter degradation capacity and can synergize with bacteria to promote soil carbon cycling (Prashar et al., 2014). After treatment with *Bacillus subtilis* 8-32, the structure of the soil fungal community changed accordingly, and the relative abundance of specific fungal taxa exhibited differential distribution characteristics. The detected abundance of some fungal taxa decreased, while fungal taxa involved in organic matter decomposition were uniquely identified. Co-occurrence network analysis revealed that the bacterial network complexity increased, whereas the fungal network simplified with a higher proportion of positive correlations. This indicated that *Bacillus subtilis* 8–32 constructs a more stable rhizosphere microecological barrier by promoting synergistic interactions among beneficial bacteria and inhibiting interspecific associations of harmful fungi (Fan et al., 2020b). The core of this regulatory mechanism lies in the strain’s selective enrichment of beneficial functional taxa rather than altering the overall community diversity, which reflects its environmental safety and ecological compatibility in agricultural applications.

Related studies have shown that under salt stress conditions, the application of *Bacillus subtilis* enhances the abundance of dominant bacterial species involveed in soil denitrification and organic matter decomposition, helping to reduce excess nitrates by converting them into nitrogen gas, thereby alleviating salt stress though oxygen consumption (Xiao et al., 2024). Moreover, *Bacillus subtilis* has been shown to improve soil water retention, promote soil desalination, and enhance cotton growth indices, while significantly increase the abundance of major bacterial phyla, including *Proteobacteria* and *Firmicutes* (Bi et al., 2024). Zhao et al. (2022) also observed changes in soil fungal diversity when applying *Bacillus subtilis* NCD-2 to suppress cotton wilt disease. The relative abundance of Ascomycetes in the treatment group was significantly higher than in the control group, with increases in the abundances of *Mortierella*, *Aspergillus*, and *Rhizopus*. The results of soil microbial diversity in this study align with trends observed in previous research, underscoring the consistency and reliability of these findings.

## Conclusion

In this study, a GFP-labeled *Bacillus subtilis* 8–32 strain was successfully constructed. This strain exhibited genetic stability, and on the basis of retaining its original growth characteristics, IAA synthesis capacity and antagonistic activity against pathogenic fungi, it could efficiently colonize tomato roots and rhizosphere soil.

*Bacillus subtilis* 8–32 exerted a growth-promoting effect on tomato plants, with the root drenching treatment (T2) exhibiting the optimal performance. Meanwhile, it significantly improved root activity and reduced MDA content in tomato seedlings, indicative of improved stress tolerance in host plants.

The strain exerted a selective regulatory effect on the tomato rhizosphere microbial community: it significantly enriched beneficial bacterial taxa such as nitrogen-fixing bacteria and organic matter-degrading bacteria (e.g., *Brucella* and *Thermomonosporaceae*), reduced the abundance of pathogenic fungi including Basidiomycota, increased the complexity of bacterial networks, simplified fungal network structure, and optimized rhizosphere microecological functions.

These findings confirm that *Bacillus subtilis* 8–32 possesses efficient colonization capacity, remarkable growth-promoting activity, and the ability to mediate targeted rhizosphere microecology regulation. Its application via root drenching shows good prospects in tomato production, providing a new approach for reducing reliance on chemical fertilizers and pesticides and promoting the development of sustainable agriculture.

## Data Availability

The raw sequencing data generated for this study has been deposited in the National Center for Biotechnology Information (NCBI) database under accession number PRJNA1353837.
